# Melatonin in Heart Failure: A Promising Therapeutic Strategy?

**DOI:** 10.3390/molecules23071819

**Published:** 2018-07-22

**Authors:** Frederic Nduhirabandi, Gerald J. Maarman

**Affiliations:** Cardioprotection Group, Hatter Institute for Cardiovascular Research in Africa (HICRA), Department of Medicine, Faculty of Health Sciences, University of Cape Town, Cape Town 7935, South Africa; gmaarman@sun.ac.za

**Keywords:** cardiac remodeling, cardioprotection, cardiomyopathy, fibrosis, heart failure, hypertension, ischemic heart disease, melatonin, metabolic syndrome

## Abstract

Heart failure is a multifactorial clinical syndrome characterized by the inability of the heart to pump sufficient blood to the body. Despite recent advances in medical management, poor outcomes in patients with heart failure remain very high. This highlights a need for novel paradigms for effective, preventive and curative strategies. Substantial evidence supports the importance of endogenous melatonin in cardiovascular health and the benefits of melatonin supplementation in various cardiac pathologies and cardiometabolic disorders. Melatonin plays a crucial role in major pathological processes associated with heart failure including ischemic injury, oxidative stress, apoptosis, and cardiac remodeling. In this review, available evidence for the role of melatonin in heart failure is discussed. Current challenges and possible limitations of using melatonin in heart failure are also addressed. While few clinical studies have investigated the role of melatonin in the context of heart failure, current findings from experimental studies support the potential use of melatonin as preventive and adjunctive curative therapy in heart failure.

## 1. Introduction

Heart failure (HF) is a complex clinical syndrome characterized by the inability of the heart to pump sufficient blood to the body due to structural and/or functional cardiac abnormalities [1]. It is commonly indicated by a reduced cardiac output and/or an elevated intracardiac pressure at rest or during exercise [1]. This syndrome affects more than 37.7 million people worldwide, and this prevalence is increasing [2,3]. Importantly, despite recent advances in HF medical care and management, its related healthcare expenses and poor outcomes in patients remain very high [2,3]. Though many factors may play a role, the increase in the global burden of HF is mostly attributed to the aging population, increased survival following acute myocardial infarction, and the high prevalence of metabolic disorders (obesity and diabetes) and related cardiac complications [1,3]. In this regard, several diseases including myocardial infarction, hypertension, certain infectious diseases (e.g., rheumatic heart disease and Chagas disease), endocrine disorders (e.g., diabetes), and cardiotoxicity (e.g., during chemotherapy and drug abuse), alone or in combination, may initiate a primary physiopathological process (e.g., oxidative stress, apoptosis and fibrosis) that leads to reduced ventricular function and, subsequently, to HF [1,3,4]. However, the mechanisms underlining the development of HF are multiple, complex, and not well understood. Current HF therapy focuses on symptomatic relief and is still elusive [1,5], and this, therefore, suggests a need for novel and effective therapeutic strategies in HF [2].

The role of melatonin (*N*-acetyl-5-methoxytryptamine) in human health and disease has become an important subject of investigation in cardiovascular research [6,7,8,9,10,11,12]. Melatonin is a small indoleamine molecule mainly produced by the pineal gland upon the activation of the suprachiasmatic nucleus of the hypothalamus during the night under normal physiological conditions (for more details on melatonin secretion and localization, see [13,14,15,16]). It exerts its traditional role as a chronobiotic or endogenous synchronizer regulating seasonal and circadian rhythms along with its sleep-inducing effects [13,16]. Also, as a multifunctional molecule, it induces numerous biological activities having potent antioxidant, anti-excitatory, anti-inflammatory, immunomodulatory, vasomotor and metabolic proprieties (for more details, see [17,18]). Of note, endogenous melatonin plays a critical role in various cardiovascular pathologies and metabolic disorders that may lead to HF [6,7,19,20,21].

The influence of melatonin on the cardiovascular system is well established [22,23,24,25,26]. Melatonin interacts with the heart and blood vessels indirectly via the nervous system and hormonal interactions [19,22,24], and directly, through its receptor-dependent and independent activities as a signaling molecule and a free radical scavenger, respectively [25,27]. Melatonin receptors comprise membrane receptors type 1 (MT1 or Mel1A or MTNR1A) and type 2 (MT2 or Mel1B or MTNR1B) which are G-protein coupled receptors, and the retinoid-related orphan nuclear receptor (RZR/RORα) [17,25]. These receptors mediate various regulatory activities of melatonin in the heart and the blood vessels [25]. Their downstream signaling effector mechanisms include adenylate cyclase, protein kinase C (PKC), phospholipase C, phospholipase A2, potassium channels, guanylyl cyclase and calcium channels and mediate the anti-adrenergic effects of melatonin [25,28,29]. In the context of HF, melatonin receptors play a significant role in the prevention of HF following myocardial infarction [29,30,31,32,33], and cardiomyopathy [20,34,35,36].

Recent studies report the beneficial effects of melatonin treatment in various animal models of HF [20,21,31,32,33,37,38,39,40,41,42]. In these models, melatonin reverses major pathological processes associated with HF including oxidative stress, apoptosis, necrosis, fibrosis and pathological remodeling [20,32,33,35,36,37,38,41,42,43]. However, given the complex etiological aspects of HF, the role of melatonin in HF is not yet well understood. The present paper discusses available evidence on the role of melatonin in ischemic and non-ischemic HF. Considering the significant role of metabolic disorders in HF, the role of melatonin in metabolic syndrome-related HF is also summarized.

## 2. Melatonin and Heart Failure: Clinical Evidence

Melatonin plays a crucial role in human cardiovascular health and disease [6,7,8,11]. Several studies demonstrate the role of endogenous melatonin in cardiovascular health and the benefits of melatonin supplementation [6,7,8,19,44]. Epidemiological studies show that pineal melatonin secretion as well as circulating melatonin levels are reduced in patients with acute and chronic HF [45,46]. Emerging studies suggest serum melatonin levels as a useful marker for HF [47]. In this context, serum melatonin levels negatively correlate with the levels of the *N*-terminal pro-brain natriuretic peptide (NT-pro-BNP), a well-known biomarker of HF [46,48]. Also, melatonin levels predict the left ventricular remodeling after acute myocardial infarction [49] and HF in patients with hypertensive cardiomyopathy [47]. Interestingly, serum melatonin levels are also associated with reverse remodeling after cardiac resynchronization therapy in patients with HF and ventricular dyssynchrony [50], therefore supporting the essential role of endogenous melatonin in HF conditions.

Melatonin treatment is considered to be a potential adjunctive chronotherapy in ischemic and hypertensive heart diseases [6,7,51,52,53]. Administration of melatonin normalizes the circadian rhythm of blood pressure and ameliorates nocturnal hypertension in hypertensive men and women receiving antihypertensive treatment [51,52], even at very old age [6,54]. Additionally, it improves the left ventricular function in HF patients with reduced ejection fraction [55]. These findings demonstrate the benefits of melatonin in HF. Besides ischemic and hypertensive heart diseases, various cardiac pathologies such as cardiomyopathy, rheumatic heart disease, cardiopulmonary disease, and congenital heart disease, either alone or in concert with other risk factors, may also lead to HF [56]. However, the clinical aspect of melatonin in these pathologies is still not yet explored.

## 3. Melatonin and Heart Failure: Experimental Evidence

### 3.1. Melatonin and Ischemic Heart Failure

Ischemic heart disease is a major underlying pathogenic factor in HF [3]. It is caused by an imbalance between cardiac blood supply, and myocardial oxygen and nutritional requirements that leads to myocardial ischemia [57]. This is also associated with the inadequate removal of metabolic end products [57] and leads to cardiomyocyte loss (via necrosis, necroptosis, apoptosis or autophagy) followed by reparative fibrotic healing, ventricular remodeling, and, eventually, HF [31,32,33,38]. Several experimental studies report the effect of melatonin in various models of ischemic HF such as post-infarction HF induced by the left anterior descending coronary artery ligation [31,32,33,37,38], chronic intermittent hypoxia-induced HF [39] and isoproterenol-induced myocardial infarction [41,58]. Collectively, melatonin treatment reduces cardiomyocyte loss and alleviates post-myocardial infarction cardiac remodeling and dysfunction in the ischemic HF models (see Table 1).

In a rat model of ischemic HF induced by the ligation of the left anterior descending coronary artery, melatonin treatment (10 mg/kg/day for four weeks) reverses all functional and biochemical defects in HF [37,38]. These benefits are associated with increased activities of cardiac sodium/ potassium-ATPase and sarcoendoplasmic reticulum calcium-ATPase (SERCA), glutathione (GSH) contents and caveolin-3 levels, and decreased activities of plasma lactate dehydrogenase, creatine kinase, lysosomal enzyme, and cardiac myeloperoxidase and malondialdehyde (MDA) [38].These cardioprotective effects of melatonin are attributed to its ability to reduce collagen and glycosaminoglycans deposition, and oxidative stress [37,38].

Additional studies in mice models of post-infarction HF demonstrate that melatonin confers cardioprotection by upregulating autophagy, decreasing apoptosis, and modulating mitochondrial biogenesis [31,32,33]. At a molecular level, melatonin activates adenosine monophosphate-activated protein kinase (AMPK) and upregulates the expression of the peroxisome proliferator-activated receptor gamma coactivator 1-alpha (PGC-1α) and the translocase of the outer mitochondrial membrane (Tom 70) receptor [31,32,33]. These effects are mediated by melatonin receptors and involve the activation of Notch1/mitochondrial fusion-associated protein 2 (Mfn2) [31], and the mammalian ste20-like kinase 1 (Mst1)/ silent information regulator 1 (Sirt1) signaling [33].

In a rat model of chronic intermittent hypoxia-induced HF, melatonin (10 mg/kg/day per four weeks) reduces myocardial inflammatory cytokines, fibrotic markers, and mitigates abnormalities in cardiomyocyte calcium-homeostasis during ischemia-reperfusion [39]. Melatonin achieves these effects by reducing calcium overload, increasing sarcoplasmic reticulum calcium content and the expression/activity of calcium-handling proteins [39]. These protective effects are accompanied by a reduction in hypoxia-induced myocardial susceptibility to ischemic injury and increased expression of antioxidant enzymes [39], and, therefore, confirm the above benefits of melatonin treatment in ischemic HF. 

Interestingly, in a rat model of acute myocardial infarction, melatonin pre-treatment (5 uM) enhances the viability of engrafted adipose tissue-derived mesenchymal stem cells (ADCS) and improves their beneficial effects in HF [59]. In this context, another study shows that melatonin treatment significantly promotes both the differentiation and the maturation of mouse embryonic stem cells to cardiomyocytes, by increasing the relative gene expression of cardiac development markers [60]. Though these data warrant further studies, they suggest the use of melatonin a practical strategy to improve the benefits and application of stem cell-based therapy for effective myocardial repair in HF [40,59,60]. 

### 3.2. Melatonin and Non-Ischemic Heart Failure

Non-ischemic HF refers to idiopathic dilated cardiomyopathy, myocarditis, alcoholic cardiomyopathy, cardiac dysfunction, and hypertensive heart disease [61]. These arise due to various primary pathological causes/stimuli that include, among others, arterial hypertension, aortic stenosis, and pulmonary hypertension [62]. If, for example, the primary pathological stimulus is arterial hypertension, it causes volume overload that leads to non-ischemic HF [62]. Whereas, if pulmonary hypertension is the primary pathological stimulus, non-ischemic HF is initiated by pressure overload [63,64]. The pressure overload increases ventricular afterload and induces concentric hypertrophy [65], and associates with fibrosis [62]. Volume overload increases ventricular preload, induces eccentric hypertrophy [65] and extracellular matrix degradation [62]. The underlying mechanism of non-ischemic HF comprises neurohormonal activation, complex metabolic changes and increased production of reactive oxygen species and oxidative stress (for reviews, see [66,67]). Given its multiple regulatory functions with potent antioxidant properties [27,68], melatonin is considered to be a potential therapy in HF [21].

**Figure 1:**
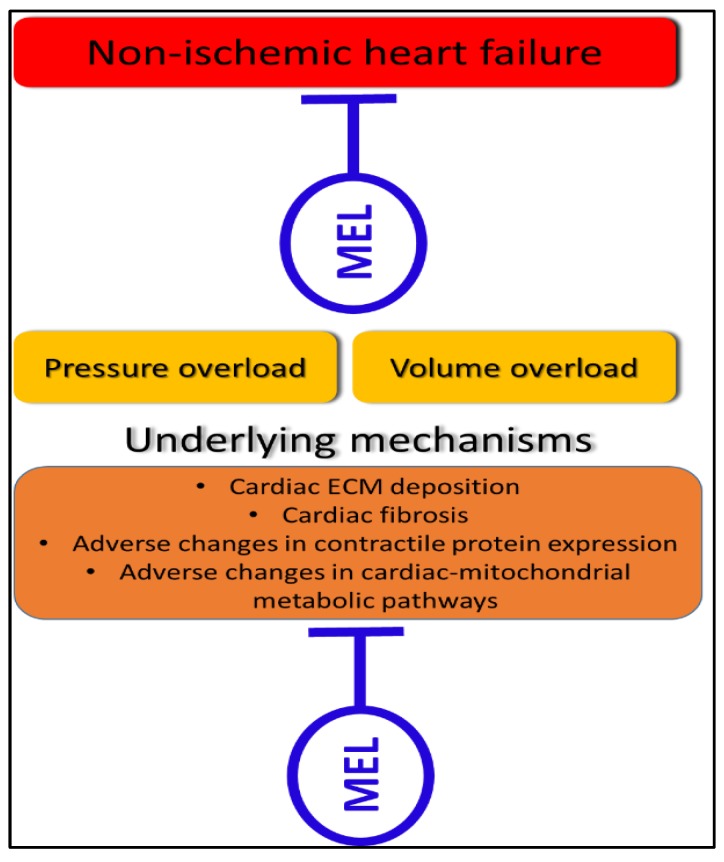
A representation of the two types of non-ischemic HF (pressure overload and volume overload), and their underlying mechanisms. This figure also depicts that melatonin can directly inhibit these underlying pathological mechanisms to confer cardioprotection in non-ischemic HF. Mel: melatonin, ECM: extracellular matrix.

At a cellular level, the main mechanistic hallmarks of non-ischemic HF include extracellular matrix deposition and fibrosis [69], adverse changes in contractile proteins [70], and alterations in cardiac mitochondrial metabolic pathways [71] (Figure 1). In the context of cardiac fibrosis, Hu and co-workers describe how effector cells are activated and cause extracellular matrix (ECM) deposition (glycosaminoglycan and collagen) in the myocardium [72]. If this deposition is moderate, it can aid in post-injury cardiac recovery, but if the deposition is excessive, it promotes myocardial scarring and HF [72]. Currently, there is no effective anti-fibrotic treatment. Interestingly, several studies show that melatonin protects the failing heart by reducing extracellular matrix deposition and fibrosis [21,73,74,75,76]. Melatonin reduces cardiac fibrosis by reducing the concentration and content of insoluble/total cardiac collagen [74,75]. This anti-fibrotic effect may be mediated via an angiotensin-II, growth factors and an angiotensin receptor pathway [21]. Therefore, by modulating the underlying mechanism of extracellular matrix deposition and fibrosis, melatonin directly stunts non-ischemic HF (Figure 1). Other mechanisms of the effects of melatonin in non-ischemic HF models are summarized in Table 2.

Besides fibrosis and ECM deposition in non-ischemic HF, the expression of contractile protein myosin heavy chain (MHC) changes from the alpha to the beta isoform [79], and this promotes non-ischemic HF [80,81]. Melatonin upregulates the expression level of α-myosin heavy chain and downregulates the expression level of the β-myosin heavy chain in non-ischemic HF [73]. Thus, this action of melatonin slows the deterioration of cardiac contractile function caused by permanent pressure overload [73]. These improvements in contractile protein expression are also associated with reduced left ventricular ejection fraction, fractional shortening, and interventricular septal thickness in diastole [73]. Consequently, melatonin directly ameliorates cardiac contractile protein expression that results in improved non-ischemic HF despite a permanent pathological stimulus (Figure 1).

Currently, mitochondrial function is regarded as a promising therapeutic target in HF [5]. The changes in cardiac-mitochondrial metabolic pathways, together with the changes in mitochondrial function and biogenesis [82] contribute to non-ischemic HF [83]. In diseases such as pulmonary hypertension, the myocardium is exposed to lower than usual oxygen that causes a shift in cardiac metabolic pathways. In this regard, cardiac metabolism shifts from oxidative phosphorylation to glycolysis, with the latter being unable to sustain cardiac functional demands during progressed cardiac disease (for review see, [84,85]). In the transverse aortic constriction model, the failing myocardium displays a reduced fatty acid oxidation and increased glycolysis pathways [86,87], and, in the pulmonary artery banding model, it displays an impaired glucose oxidation and a decreased energy reserve, with a subsequent insufficient energy supply [88]. Melatonin is known to improve cardiac-mitochondrial function and metabolism [89]. However, the effect of melatonin in such metabolic pathways in non-ischemic HF remains poorly described. Therefore, future studies could investigate the impact of melatonin on cardiac-mitochondrial metabolic pathways in non-ischemic HF. These metabolic pathways can be assessed with high-resolution respirometry that can assess the capacity of cardiac-mitochondrial electron transfer system, proton leakage, and electron transfer system-complex activities. Such investigations can make essential contributions to the body of knowledge regarding the role of melatonin in non-ischemic HF.

Mitochondrial biogenesis plays a crucial role in non-ischemic HF [5]. Non-ischemic HF induced by pulmonary artery banding [90] or transverse aortic constriction [91] is associated with a reduction in cardiac-mitochondrial biogenesis. Primary pathological stimuli in non-ischemic HF alter critical proteins that result in a downregulation of cardiac-mitochondrial biogenesis [82]. This mechanism is believed to contribute to the failing myocardium [82]. Zhai and co-workers were the first to investigate the effects of melatonin on cardiac-mitochondrial biogenesis in non-ischemic HF [73]. Using an in vivo transverse aortic constriction-induced pathological cardiac hypertrophy model, they demonstrated that melatonin upregulates the expression of peroxisome proliferator-activated receptor gamma coactivator-1 beta (PGC1-β, a proxy for mitochondrial biogenesis) in non-ischemic HF [73]. These results suggest that even in non-ischemic HF, where the primary pathological stimulus is permanent, melatonin can promote cardiac-mitochondrial biogenesis. It is, therefore, likely that in this model, melatonin stimulates an adaptive response to the primary pathological stimulus [73]. This comprises the upregulation of cardiac-mitochondrial biogenesis as reflected by the increased expression of PGC1-β [73]. The increased number of mitochondria may aid the failing myocardium in producing sufficient energy to maintain cardiac energy requirements in HF [82,92]. These findings support the argument that melatonin directly and beneficially upregulates cardiac-mitochondrial biogenesis, to improve non-ischemic HF (Figure 1). The role of mitochondria in the protective effects of melatonin may be explained by the highest concentration of melatonin in the mitochondria as compared to the other subcellular compartments [14], and the recently discovered production and secretion of melatonin by mitochondria [93,94,95].

## 4. Role of Melatonin in Heart Failure Related to the Metabolic Syndrome

Features of the metabolic syndrome include high blood pressure, insulin resistance, lipid abnormalities, diabetes, and obesity [96]. These features and related diseases such as obstructive sleep apnea, are highly prevalent in HF patients and play a critical role in the progression from subclinical to clinical ventricular dysfunction and HF (for review, see [96,97]). Multiple molecular and cellular responses including neurohormonal activation, complex metabolic changes and increased production of reactive oxygen species, and oxidative stress contribute to the development of HF in metabolic syndrome [96]. Melatonin has recently received attention as a potential therapy in obesity and related cardiometabolic abnormalities [17,34,44,98,99,100]. Its role in metabolic syndrome is reported in both animal and human studies [17,34,44,98].

In pre-diabetic animal models, short-term melatonin treatment (4 mg/kg per day for three weeks) protects the hearts of diet-induced obese rats, independent of body weight and fat mass [101]. Whereas, long-term melatonin administration reverses the metabolic abnormalities associated with insulin resistance and dyslipidemia and protects the hearts of the obese rats [102]. In these animals, melatonin treatment (4 mg/kg per day for six weeks) also increases basal and insulin-stimulated glucose uptake by cardiomyocytes isolated from the hearts of obese, insulin-resistant rats, supporting the insulin-sensitizing effect by melatonin [100]. Impairment of insulin-stimulated glucose uptake is considered the most consistent change that develops early in insulin-resistant hearts [103]. It also associates with increased oxidative stress and cardiomyopathy [104,105]. Interestingly, a recent study in the DahlS.Z-Lepr^(fa)^/Lepr^(fa)^ (DS/obese) rat model of metabolic syndrome, shows that melatonin receptor agonist, ramelteon treatment at either low (0.3 mg/kg per day) or high (8 mg/kg per day) dose attenuates body weight gain, left ventricular fibrosis, and diastolic dysfunction, as well as cardiac oxidative stress and inflammation [34]. Similar beneficial effects on cardiac hypertrophy and fibrosis are also reported in pre-diabetic obese (ob/ob) mice treated with melatonin (100 mg/kg per day in drinking water) for 8 weeks (from 5 weeks of age) [99]. In this model, melatonin induces its beneficial effects by reversing the mitochondrial and metabolic defects in the hearts [99]. These findings support the importance of melatonin and the potential use of melatonin receptor agonists in HF-related metabolic syndrome.

In a diabetic rat model, melatonin also ameliorates metabolic risk factors including lipid abnormalities, insulin resistance, modulates apoptotic proteins, and protects the heart against diabetes-induced apoptosis and cardiomyopathy [104]. This finding suggests the use of melatonin as a preventive approach against HF in patients with the metabolic syndrome. This beneficial effect of melatonin in diabetic rats is also reported in other diabetic models [20,35,43], and it is further supported by recent clinical studies [106,107]. As the underlying mechanism, melatonin alleviates cardiac remodeling and dysfunction in the diabetic heart by upregulating autophagy and limiting apoptosis while modulating mitochondrial integrity and biogenesis [20]. These effects are mediated by various intracellular signaling pathways including Mst1/silent information regulator 3 (Sirt3) [20], dynamin-related protein 1 (Drp1)/Sirt1 [43] and spleen tyrosine kinase (Syk) [35].

Clinical studies show that reduced melatonin secretion levels are associated with a higher risk of incident myocardial infarction in women with increased body mass index [108]. These findings suggest that melatonin may be an effective therapy in obesity-related abnormalities that may predispose patients to ischemic HF. For example, melatonin supplementation (5 mg/day, two hours before bedtime, for two months) improves blood pressure, lipid profile, and parameters of oxidative stress in patients with metabolic syndrome [107]. It is well-known that the presence of diabetes per se adversely affects long-term survival and risk of hospitalization in patients with acute and chronic HF [109]. Interestingly, in a randomized, double-blind, placebo-controlled trial involving 60 diabetic patients with coronary heart diseases melatonin (10 mg once a day for 12 weeks) exerts its beneficial effects by ameliorating serum C-reactive protein levels, glycemic control, and high-density lipoprotein-cholesterol [106]. Given the high prevalence of diabetes in HF patients, the promising beneficial effects of melatonin should be explored for future effective therapy in HF patients with metabolic syndrome.

## 5. Current Challenges and Perspectives in the Use of Melatonin in Heart Failure

Even though the use of animals has provided more insight into the pathophysiological mechanisms of HF and the development of new therapies, the complex etiology of HF still makes it challenging to study HF using animal models [110]. Melatonin was suggested as a preventive and curative therapy against various forms of the cardiac disease [111,112]. However, in the context of HF, there are a few important things to consider. A reciprocal relationship exists between the anatomic changes of the myocardium and the initial pathological stimulus (e.g., pulmonary hypertension) [63]. This means that treatment with melatonin may directly modulate the primary pathological stimulus (local effects) or can be released into circulation (systemic effects) where it affects the remodeled myocardium. In a rat model of pulmonary hypertensive rats, melatonin confers cardioprotection and stunts non-ischemic HF [21]. Even in this model, the protective effects of melatonin may either be due to its direct effect on the remodeled myocardium or the primary pathological stimulus; it is likely that melatonin simultaneously induces beneficial effects against the primary pathological stimulus and on the remodeled myocardium. This is especially relevant in studies where melatonin treatment is administered via drinking water or oral pills formations. In such models, melatonin is released into systemic circulation and may thereby have beneficial effects on the remodeled myocardium and the initial pathological stimulus [21].

In most models of non-ischemic HF, it is challenging to determine whether melatonin can directly interfere with the non-ischemic HF process. If one wants to delineate the effects of melatonin on the actual cardiac remodeling/HF process, a more appropriate model would be transverse aortic constriction or pulmonary artery banding [110]. With this model, a silk suture is permanently placed on the aorta, or an occluding hemoclip is permanently placed on the pulmonary artery trunk [110]. Thus, there is an artificial primary pathological stimulus that cannot be changed or altered by melatonin, yet non-ischemic HF remains present. Such models would authenticate a claim that melatonin can interfere (beneficially) with the non-ischemic HF process. In line with this notion, the effects of melatonin were recently studied in an in vivo model of transverse aortic constriction-induced HF [73]. Despite the permanent silk suture (unchangeable artificial, primary pathological stimulus); melatonin stunted non-ischemic HF [73]. This observation confirms that melatonin can, in fact, stunt the HF process, even when the permanent pressure overload caused by the suture remains permanent/unchanged.

In the context of metabolic syndrome, melatonin secretion levels are low in patients with insulin resistance and impaired glucose tolerance [113], and mutations in MTNR1B gene are associated with an increased risk of diabetes [114,115]. Moreover, the nuclear melatonin receptor RORα is down-regulated in the diabetic heart, and its deficiency aggravates the diabetic cardiomyopathy and HF [36]. While administration melatonin or RORα agonist reduces cardiomyocyte hypertrophy and fibrosis and improves cardiac function [36], a direct need for an exploration of the benefits of melatonin for the prevention of HF in diabetic states warrants further studies. Hypothetically, given the potent antioxidant and anti-inflammatory properties of melatonin, its administration may be beneficial in diabetic people with HF [116]. However, previous reports on the role of melatonin on glucose homeostasis are inconsistent [117], and mutations in MTNR1B in diabetic patients may further worsen the deleterious effect of melatonin on glucose tolerance in humans [118,119]. This observation requires a careful consideration for eventual personalized use of melatonin in HF patients with diabetes.

Melatonin treatment is also beneficial in other models of HF-related cardiomyopathies that are not explicitly described in this paper. For example, it protects against cardiomyopathy induced with ovariectomy [120], radiation [121], hyperthyroidism [105], chemotherapies [76,122], aluminum phosphide [123], lipopolysaccharides and sepsis [42,124,125], and chronic Chagas disease [126]. Thus far, melatonin is not yet studied in animal models of peripartum cardiomyopathy, another type of non-ischemic HF. Future study in this regard may uncover exciting results given the involvement of prolactin in the pathology of the peripartum cardiomyopathy, and the interference of prolactin and melatonin in the processes responsible for the development and maintenance of pregnancy [127].

Additional challenges and limitations to the use of melatonin in cardiovascular diseases were recently summarized somewhere else [7]. As previously highlighted, the current challenge for using melatonin is mostly its dosage, low bioavailability, and unknown long-term effect of high doses [7,128]. The use of melatonin as a nutritional supplement is widely accepted [129]. The dietary melatonin and phytomelatonin represent an alternative for effective melatonin preventive treatment [7]. However, the lack of standardized methods to determine melatonin concentration in foods or in plants, together with the low melatonin bioavailability following oral administration in humans (approximately 15%) with, moreover, a short maximal half-life [128], makes hard to attribute some of the observed benefits to the dietary melatonin [7,130]. Most of the melatonin-rich foods might also have other cardioprotective ingredients [131]. Nevertheless, the increase in circulating melatonin following the consumption of melatonin-rich food may be itself a good indication of the potential effects of melatonin [132].

Despite multiple cardiovascular benefits of melatonin supplementation, it is worth mentioning that due to various reasons few studies do not demonstrate the protective effect of melatonin in HF, such as in a rat model of isoproterenol-induced left ventricular hypertrophy [133] and a rabbit model of myocardial infarction [134]. This observation is consistent with the recent clinical studies conducted in the context of myocardial ischemia/reperfusion injury where the effect of melatonin administration is neutral [135,136] with unexpected detrimental effect favoring ventricular remodeling [135]. Most of the unexpected findings are due to methodological issues including, mainly, the severity of cardiac damage, dosages, time and mode of administration of the treatment, the type of animal models, age and comorbidities [137]. Nevertheless, all of these studies confirm the safety of melatonin treatment [133,134,135]. Accordingly, better-designed studies are needed to delineate the role of melatonin in HF and related conditions. 

In addition, besides the well-documented safety of melatonin [134,135,138], other reports show that circulating melatonin levels are high in some HF patients [139], and its supplementation may also be detrimental [139,140,141]. These data suggest that melatonin should be used with caution in humans. However, in view of the multiple cardiovascular benefits of melatonin [8,53], these studies present a very little substantive evidence to support any significant adverse effects of melatonin at the level of the heart. Melatonin is an endogenously produced molecule and is also consumed in edible plants and other foodstuffs, and its safety is well documented [138]. As recently stated, its multiple benefits at the cost of very few side effects may exceed those of some drugs much more widely used for cardioprotection [142].

The exploration of the role of melatonin in HF is clinically relevant. Melatonin is currently prescribed for the regulation of sleep patterns such as in the jet lag [143] and sleep disorders [144]. However, very few clinical studies have thus far investigated the beneficial effects of melatonin supplementation in patients with HF [11]. Considering the correlation between circulating melatonin levels and the well-established biomarkers of HF such as NTpro-BNP, high-sensitivity C-reactive protein, and lipid peroxidation in HF patients [46,48,106], further studies are needed. These studies could determine the pathogenic as well as the prognostic importance of melatonin alterations in patients with chronic HF.

## 6. Conclusions

**Figure 2:**
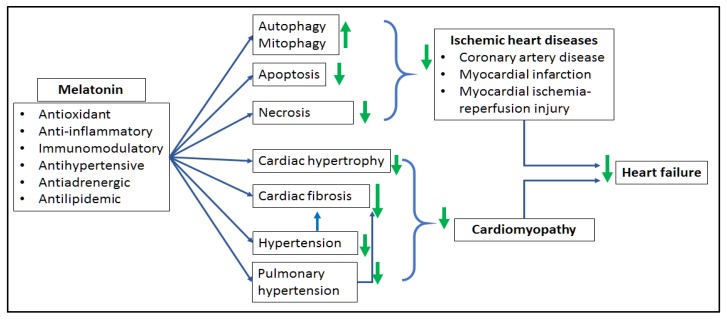
Summary of the beneficial effect of melatonin in cardiac pathologies associated with the development of heart failure. Melatonin exerts its antioxidant, anti-inflammatory and immunomodulatory properties and protects the heart against ischemic heart disease characterized by myocardial cell death (necrosis, apoptosis, autophagy/mitophagy) as well as subsequent post-infarction cardiac dysfunction and ischemic HF. Arterial hypertension and pulmonary hypertension induce both cardiac fibrosis and pathological remodeling (cardiomyopathy) with subsequent ventricular dysfunction and HF. Melatonin reverses these effects and prevents HF; ↓: increases, ↑: reduces (references in the text). Figure reproduced from [7] with permission.

As highlighted in this paper, HF is a complex clinical syndrome with two predominant etiological aspects, ischemic and non-ischemic HF. In view of the significant alterations of melatonin levels in HF patients, the exploration of the role of melatonin in HF is clinically relevant. So far, results obtained from recent preclinical and clinical studies are promising. Administration of melatonin reverses major pathological processes associated with HF including oxidative stress, apoptosis, necrosis, fibrosis and pathological remodeling (Figure 2). Though additional mechanistic studies are still required to delineate the underlying mechanisms of the effect of melatonin in HF, melatonin is an important, safe, and affordable molecule worthy to be used as preventive and adjunctive curative therapy in HF.

## Figures and Tables

**Table 1:** The effect of melatonin in animal models of ischemic heart failure.

Animal Model	Genre and Strain (Age or Weight)	Melatonin TreatmentMode, Dose and Duration	Effect of Melatonin and the Underlying Mechanism (↓: Decrease, ↑: Increase)	References
A murine model of post-infarction cardiac remodeling and dysfunction (in vivo)	Male C57BL Mice (8–12 weeks)	Oral, 20 mg/kg/day for 1 week before myocardial infarction	Cardioprotection: ↓ cardiac dysfunction; ↓ adverse left ventricle remodeling; ↑ autophagy, ↓ apoptosis, ↓ mitochondrial dysfunction, ↓ Mst1 expression and ↑ Sirt1 signaling	[33]
A murine model of myocardial infarction (ligation of the left anterior descending coronary artery for 5 days)	Male C57BL Mice (10–12 weeks)	Intraperitoneal, 10 mg/kg/day, 2 weeks before and 20 mg/kg, 3 h after myocardial infarction	Cardioprotection: ↓ post-myocardial infarction damage, ↑ peroxisome proliferator-activated receptor gamma coactivator-1 alpha (PGC-1α) and Tom 70 expression, ↑mitochondrial integrity, ↓ reactive oxygen species (ROS) production	[32]
A murine model of myocardial infarction	female C57BL/6a mice (8 weeks) (20–25 g)	5 μM for 24 h before adipose tissue-derived mesenchymal stem cells transplantation (intramyocardial injection)	Cardioprotection: ↑ Sirt1 signaling, ↑ expression of anti-apoptotic protein Bcl2, ↓ expression of acetylated-forkhead box O1 (Ac-FoxO1), acetylated-p53 (Ac-p53), Ac-NF-κB, and Bax	[40]
A murine model of myocardial infarction (in vivo)	Male C57BL Mice (10–12 weeks)	Intraperitoneal, 10 mg/kg/day, 2 weeks before and 20 mg/kg, 3 h after myocardial infarction	Cardioprotection: ↓ post- myocardial infarction damage, ↑ Notch1 signaling and Mfn2 expression via melatonin receptors	[31]
A rat model of myocardial infarction (in vivo)	Male Sprague-Dawley rats (8–10 weeks)	5 μM pre-treatment for 24 h (adipose tissue-derived mesenchymal stem cells)	Cardioprotection: ↑ antioxidant enzyme catalase and Cu/Zn superoxide dismutase (SOD), ↑ pro-angiogenic and mitogenic factors like insulin-like growth factor 1 (IGF-1), basic fibroblast growth factor (b-FGF), hepatocyte growth factor (HGF), epidermal growth factor (EGF), ↑ anti-apoptosis kinases like p-Akt, ↓ caspase cascade	[59]
A rat model of myocardial infarction-induced heart failure (in vivo)	Male Wistar albino rats (200–250 g)	Intraperitoneal, 10 mg/kg/day for 4 weeks after myocardial infarction	Cardioprotection: ↑ cardiac Na^+^, K^+^-ATPase and SERCA activities, glutathione contents and caveolin-3 levels, ↓plasma lactate dehydrogenase (LDH) and creatine kinase (CK), lysosomal enzyme activities and cardiac malondialdehyde (MDA) and Myeloperoxidase (MPO)	[38]
A rat model of isoproterenol-induced myocardial infarction (in vivo)	Sprague-Dawley rats (10 weeks, 175–225 g)	Intraperitoneal, 10 mg/kg/day for 7 days	Cardioprotection: ↓ cardiac injury markers (creatine kinase-MB, lactate dehydrogenase, aspartate transaminase and alanine transaminase), ↑ cardiac antioxidant defense system, normalizes lipid profile in the serum and heart tissue	[58]

**Table 2:** The effect of melatonin in animal models of non-ischemic heart failure.

Animal Model	Genre and Strain (Age or Weight)	Melatonin Treatment (Mode, Dose and Duration)	Effect of Melatonin and the Underlying Mechanism (↓: Decrease, ↑: Increase)	References
A murine model of pathological cardiac hypertrophy (induced by transverse aortic constriction) (in vivo)	Male C57BL/6 mice (20–25 g) (8–10 weeks)	Oral, 20 mg/kg/day for 4 or 8 weeks	Cardioprotection: ↓ pulmonary congestion, ↓ cardiac fibrosis, ↓ the deterioration of cardiac contractile function (↑ expression of the α-myosin heavy chain, ↓ expression of β-myosin heavy chain), ↓ atrial natriuretic peptide, ↑ expression of peroxisome proliferator-activated receptor-gamma coactivator-1 beta (PGC-1 β), ↓ oxidative stress	[73]
A rat model of hypoxic pulmonary hypertension with intermittent chronic hypoxia for 4 weeks (in vivo)	Male Sprague-Dawley rats (200–250 g)	Intraperitoneal, 15 mg/kg/day, morning for 1 week before hypoxia and during hypoxia (4 weeks)	Cardioprotection: ↓ right ventricular systolic pressures (RVSP), ↓ weight of the right ventricle/left ventricle plus septum (RV/LV+S) ratio, ↓ pulmonary vascular structure remodeling; ↓ proliferating cell nuclear antigen (PCNA), ↓ hypoxia-inducible factor-1alpha (HIF-1α), ↓ nuclear factor-kB (NF-kB), ↓ proliferation of primary pulmonary artery smooth muscle cells (PASMCs), ↓ phosphorylation of Akt, ↓ extracellular signal-regulated kinases1/2 (ERK1/2)	[77]
Chronic intermittent hypoxia, model of a severe obstructive sleep apnea for 2 to 3 weeks (in vivo and ex vivo)	Adult Sprague-Dawley rats	Intraperitoneal, 10 mg/kg/day at 30 min before hypoxic exposure	Cardioprotection: ↓ blood pressure (BP), ↓ oxidative stress, endothelial dysfunction, and inflammation: ↑ MDA, expressions of nicotinamide adenine dinucleotide phosphate (NADPH) oxidase, pro-inflammatory mediators (TNF-α, inducible NO synthase, COX-2), ↓ cellular adhesion molecules, ↑ nitric oxide (NO˙), endothelial-dependent relaxation, endothelial NO synthase (eNOS), antioxidant enzymes (catalase (CAT), glutathione peroxidase-1 (GPx), Cu/Zn Superoxide dismutase (SOD))	[78]
A rat model of isoproterenol-induced HF (in vivo)	Male Wistar rats (3 months)	Oral, 10 mg/kg/day for 2 to 4 weeks	Cardioprotection: ↓ cardiac fibrosis but with no effect on the right ventricle/left ventricle (LV/RV) hypertrophy; ↓ oxidative stress, insoluble and total collagen, the beta-tubulin alteration in the LV	[75]
Monocrotaline (MCT)- induced pulmonary hypertensive rats (ex vivo cardiac function)	Male Long Evans rats (150–175 g)	Oral, 75 ng/L; 6 mg/kg/day or 2 or 4 weeks, preventive at 5 days before MCT for 4 weeks or curative at 2 weeks after MCT for 2 weeks	Cardioprotection (curative and preventive): ↓ right ventricle (RV) hypertrophy, ↑ RV-function, ↓ systemic oxidative stress, ↓ cardiac interstitial fibrosis	[21]
Continuous light-induced hypertensive rats for 6 weeks (in vivo)	Male Wistar rats (3 months)	10 mg/kg/day, Oral for 6 weeks	Cardioprotection: ↓ cardiac fibrosis, oxidative stress, but with no effect on LV hypertrophy	[74]
A rat model of metabolic syndrome- induced cardiac injury (in vivo)	DahlS.Z-Leprfa/Leprfa (DS/obese) rats (8 weeks)	Melatonin receptor agonist (ramelteon) at a low (0.3 mg/kg per day) or high (8 mg/kg per day) dose from 9 weeks of age, for 4 weeks	Cardioprotection: ↓ body weight gain, left ventricular fibrosis, and diastolic dysfunction, cardiac oxidative stress and inflammation, ↑ insulin signaling in visceral and subcutaneous white adipose tissue; ↓ mitochondrial uncoupling protein 1 (UCP-1), ↓ whitening of brown adipose tissue	[34]
